# Securing the Achilles’ Heel of Esophagectomy: An Updated Evidence-Based Roadmap for Anastomotic Leak Prevention

**DOI:** 10.3390/cancers18081294

**Published:** 2026-04-19

**Authors:** Lorenzo Viggiani d’Avalos, Marcel A. Schneider, Diana Vetter, Pascal Burri, Daniel Gerö, Christian A. Gutschow

**Affiliations:** Department of Visceral and Transplant Surgery, University Hospital Zurich, 8091 Zurich, Switzerland; lorenzo.viggianidavalos@usz.ch (L.V.d.); marcelandre.schneider@usz.ch (M.A.S.); diana.vetter@usz.ch (D.V.); pascal.burri@usz.ch (P.B.); daniel.gero@usz.ch (D.G.)

**Keywords:** esophagectomy, anastomotic leakage, gastric conduit, indocyanine green (ICG), preemptive endoluminal vacuum therapy (pEVT), prehabilitation, sarcopenia, RAMIE

## Abstract

**Background**: Esophagectomy remains the definitive curative treatment for esophageal cancer but is historically burdened by significant procedure-related morbidity. Anastomotic leakage (AL) is still the “Achilles’ heel” of esophageal surgery, serving as a primary benchmark for surgical quality due to its profound impact on patient recovery, healthcare costs, and long-term oncological outcomes. While surgical expertise and perioperative care have matured, reported AL rates remain persistently high. This necessitates a shift in focus from purely technical modifications toward integrated, data-driven preventive strategies. **Purpose**: Five years after our initial review, this update synthesizes the rapid evolution in AL prevention. We evaluate the transition from empirical surgical pragmatism to evidence-based protocols, integrating recent breakthroughs in real-time perfusion monitoring, prophylactic endoluminal technologies, and multidisciplinary patient optimization. This work provides a contemporary “roadmap” for navigating the complexities of esophageal reconstruction. **Conclusions**: The prevention of AL has evolved into a multimodal “bundle” that begins well before the index operation. This review highlights the critical shift toward quantitative perfusion assessment via indocyanine green fluorescence angiography, which is increasingly replacing subjective visual inspection as the standard for anastomotic site selection. We discuss the emerging role of gastric ischemic preconditioning as a biological strategy to enhance conduit vascularity, alongside the paradigm of proactive management using preemptive endoluminal vacuum therapy to mitigate septic sequelae in high-risk cases. Furthermore, we examine technical refinements in conduit construction and conditioning—focusing on the ‘tension-perfusion’ relationship—and the essential role of structured prehabilitation within enhanced recovery after surgery frameworks. While the quality of evidence remains heterogeneous, the move toward standardized reporting and objective monitoring marks a new era of precision in esophageal surgery.

## Simple Summary

Esophagectomy is a major operation used to treat esophageal cancer. However, it can be complicated by anastomotic leakage, a failure of the surgical connection between the esophagus and the stomach to heal properly. This complication can lead to severe infections, prolonged hospital stays, and worse long-term survival. Despite advances in surgical techniques, leakage rates remain relevant. In this review, we summarize recent progress in preventing this complication and propose a comprehensive “roadmap” that spans the entire treatment pathway. Key strategies include improving patients’ physical and nutritional condition before surgery, using imaging techniques during the operation to assess blood flow, and applying early supportive treatments after surgery. By combining these measures into a structured, evidence-based pathway, surgical teams can improve patient safety and recovery. These findings may guide future research and help standardize care in esophageal cancer surgery.

## 1. Introduction

Esophagectomy remains the definitive curative treatment for localized esophageal cancer; however, it continues to carry substantial procedure-related morbidity, even when performed in high-volume centers. Among the spectrum of postoperative complications, anastomotic leakage (AL) stands out as the most critical—often described as the “Achilles’ heel” of esophageal surgery. AL occurs in up to 30% of cases, with incidence rates fluctuating based on surgical technique, the anatomical location of the anastomosis, and individual patient characteristics [1,2,3].

The clinical consequences of AL are profound. Patients frequently face prolonged stays in the intensive care unit (ICU), high reintervention rates, and significantly increased postoperative mortality. Beyond these immediate harms, growing evidence suggests that AL negatively impacts long-term oncologic outcomes, including an increased risk of cancer recurrence and reduced overall survival [4]. Furthermore, survivors often suffer from impaired health-related quality of life due to persistent dysphagia, stricture formation requiring repeated dilations, and chronic psychological distress [5]. Because of this multifaceted impact, AL has become a critical surgical quality indicator for benchmarking institutional performance.

Despite significant advancements in surgical expertise and the widespread adoption of enhanced recovery after surgery (ERAS) pathways, the incidence of AL has remained relatively stable over the last decade. This persistent challenge has driven a paradigm shift away from purely technical modifications toward comprehensive, multimodal prevention “bundles” implemented throughout the entire perioperative pathway. Modern strategies now emphasize a proactive approach: beginning with preoperative risk stratification and biological optimization, followed by meticulous intraoperative techniques augmented by objective real-time perfusion assessment, and concluding with vigilant postoperative monitoring using individualized treatment algorithms. In this context, the aim of this narrative review is to synthesize the current spectrum of these strategies and evaluate their scientific evidence and clinical significance. Building upon our previously established framework for AL prevention [5], this update provides a contemporary “roadmap” for the surgical team. We focus on the four critical perioperative phases: preoperative optimization (including prehabilitation and ischemic conditioning), perioperative anesthesiologic management (focus on hemodynamics and microvascular perfusion), intraoperative technical refinements (focusing on conduit construction and perfusion technologies), and proactive postoperative management. Additionally, we address the evolving understanding of AL’s effects on oncologic outcomes and discuss novel technologies designed to move the needle on this major surgical challenge.

### Methods

This narrative review is based on a structured literature search of PubMed and Embase, focusing on studies published within the last 5–10 years, with particular emphasis on high-level evidence such as randomized controlled trials, meta-analyses, and large cohort studies. Seminal earlier studies were included where relevant. Study selection was guided by clinical relevance to perioperative anastomotic leak prevention and the aim of providing an integrative, practice-oriented overview rather than a formal systematic synthesis.

## 2. Preoperative Optimization: From Risk Stratification to Prehabilitation

The preoperative phase has evolved from simple patient selection into a proactive window of opportunity. While the fundamental risks remain, our ability to quantify and mitigate them has significantly matured over the last five years.

### 2.1. Patient-Related Risk Factors and the Shift to Physiological Age

Traditionally, comorbidities such as obesity, heart failure, COPD, and diabetes mellitus have been recognized as independent predictors of AL due to their negative impact on microvascular perfusion and tissue healing [6]. Recent evidence, however, suggests a shift in focus from chronological age to physiological resilience. While earlier data on age (≥75 years) as a risk factor remained heterogeneous, contemporary research has increasingly explored the role of sarcopenia and low skeletal muscle mass—objectively assessed using CT-based body composition analysis—as potential predictors of anastomotic leakage and postoperative morbidity [7]. However, the definition and clinical relevance of sarcopenia remain heterogeneous. In particular, the relative contribution of reduced muscle mass versus impaired functional capacity is not consistently assessed across studies, and current evidence regarding its predictive value for anastomotic leakage is inconsistent. Therefore, CT-derived sarcopenia should be interpreted as a surrogate marker of physiological reserve rather than a definitive predictor. Diabetes mellitus remains a critical modifiable factor; current data reinforces that poor glycemic control (HbA1c > 7–8%) significantly impairs wound healing. Similarly, the role of vascular calcification (aortic and coeliac trunk) as a radiological surrogate for “arteriosclerotic burden” has gained further traction, suggesting that general arteriosclerosis scores are valuable tools for estimating the “biological quality” of the future gastric conduit [8].

### 2.2. Nutritional Optimization and the Immunonutrition Debate

Malnutrition (weight loss > 10%, BMI < 18.5 kg/m^2^) remains prevalent in up to 60% of esophageal cancer patients. The ESPEN guidelines—advocating 7–14 days of preoperative support for severely malnourished patients—remain the gold standard and can reduce complications by up to 50% [9]. On the other hand, the role of immunonutrition (enriched with omega-3 fatty acids, arginine, and nucleotides) has become more nuanced. While earlier systematic reviews suggested a broad benefit in gastrointestinal surgery, more recent evidence presents a more complex picture. The multicenter double-blind randomized NEOIMMUNE trial did not demonstrate a benefit of immunonutrition administered during neoadjuvant therapy in terms of health-related quality of life or postoperative morbidity, with similarly low anastomotic leak rates observed between groups. In parallel, recent meta-analyses suggest a reduction in overall infectious complications, although the direct impact on anastomotic leak remains uncertain. Taken together, these findings support a shift toward a selective, patient-tailored use of immunonutrition rather than its routine application in all patients undergoing esophagectomy [10].

### 2.3. Prehabilitation: The Multimodal “Bundle”

One of the most significant evolutions in the last five years is the transition from isolated measures (like simple spirometry) to structured, multimodal prehabilitation programs. These programs, typically spanning 4–6 weeks, integrate:

- Physical Component: Combining inspiratory muscle training with aerobic and resistance exercise to increase functional capacity.
- Nutritional Component: Targeted protein supplementation (1.5–2.0 g/kg/day).
- Psychological Support: To reduce perioperative stress, improve treatment adherence, and address modifiable behavioral risk factors such as smoking and alcohol consumption. Structured psychological support may also enhance patient engagement and resilience during neoadjuvant treatment and the perioperative period.

Recent meta-analyses now provide stronger evidence that these structured programs can indeed reduce the incidence of AL by optimizing the patient’s physiological reserve before the “index stress” of surgery [11].

### 2.4. Tumor- and Treatment-Related Factors: The Shifting Neoadjuvant Landscape

The “Achilles’ heel” of esophagectomy is increasingly influenced by the rapid evolution of neoadjuvant and perioperative oncological protocols. While the surgical community has long focused on the impact of radiation, the widespread adoption of intensive chemotherapy and the arrival of immunotherapy have added new layers of complexity to tissue healing and conduit integrity.

Neoadjuvant chemoradiotherapy (nCRT), historically the standard of care following the CROSS trial [12,13], remains under scrutiny regarding its impact on the anastomosis. Radiation-induced fibrosis and microvascular damage—particularly when the radiation field involves the proximal stomach (the future conduit)—are theoretical risk factors for impaired healing. However, recent data suggests that these risks can be mitigated by precision in the surgical timeline. Maintaining an optimized interval of 6–8 weeks between the completion of nCRT and surgery appears to balance the benefits of tumor regression with the potential for progressive radiation-induced tissue damage [14,15]. For esophageal adenocarcinoma, the paradigm has shifted toward perioperative chemotherapy (FLOT), which avoids the potential “collateral damage” of radiation to the conduit tissue. From a surgical perspective, intensive taxane-based regimens like FLOT present a different challenge: the systemic impact on the patient’s nutritional and functional status (sarcopenia) rather than local tissue fibrosis. While FLOT does not appear to increase primary AL rates compared to nCRT, the associated systemic toxicity necessitates even more rigorous preoperative prehabilitation to ensure the patient’s “biological age” is optimized for the index stress of surgery. The most recent disruptive change is the integration of immunotherapy into the neoadjuvant and perioperative setting. The Matterhorn trial, evaluating the addition of Durvalumab to FLOT, and the established use of Nivolumab as an adjuvant treatment for patients with residual disease (CheckMate-577), marks a new era. While these checkpoint inhibitors have revolutionized oncological outcomes, their impact on anastomotic healing is still being characterized. Theoretically, the immune-mediated inflammatory response could influence the early phases of tissue repair. However, preliminary surgical safety data from these major trials are encouraging, suggesting that the addition of immunotherapy does not significantly increase the incidence of AL—provided that the surgical team remains vigilant for immune-related adverse events that could complicate the postoperative recovery [16,17].

### 2.5. Data-Driven Risk Stratification

The integration of Machine Learning (ML) models marks the newest frontier in the roadmap. Moving beyond subjective clinical judgment, these models incorporate multi-dimensional variables (patient, tumor, and treatment data) to predict AL risk [18]. While no single score has replaced clinical intuition, these tools increasingly support multidisciplinary teams in making personalized decisions—such as choosing between cervical and intrathoracic anastomosis or selecting patients for proactive postoperative interventions.

Taken together, all these factors may be integrated into a pragmatic risk stratification approach to identify high-risk patients who may benefit from intensified prehabilitation or proactive postoperative strategies such as pEVT.

## 3. Perioperative Management: Hemodynamics and Microvascular Perfusion

The perioperative phase represents a physiological “tightrope walk,” where the anesthesiologic goal is to maintain systemic stability without compromising the microcirculation of the newly created gastric conduit. Recent years have seen a transition from rigid fluid protocols toward individualized, data-driven hemodynamic management.

### 3.1. From Restrictive to Goal-Directed Fluid Therapy (GDT)

For years, restrictive perioperative fluid management was the cornerstone of esophagectomy protocols to minimize pulmonary complications and interstitial edema at the anastomotic site [19]. However, the landscape has evolved. We now recognize that excessive fluid restriction can trigger a cascade of hypovolemia and hypotension, necessitating the use of high-dose catecholamines, which in turn may induce microvascular vasoconstriction and ischemia in the conduit—the “Achilles’ heel” of the reconstruction [20,21,22].

Modern “Roadmaps” now advocate for GDT. Using advanced monitoring—such as stroke volume variation or pulse pressure variation—anesthesiologists can now tailor fluid administration to the patient’s actual preload requirements. This approach aims to maintain the “Goldilocks zone” of hydration: avoiding both the pulmonary risks of fluid overload and the ischemic risks of hypovolemia [19].

### 3.2. The Vasopressor Dilemma and Mean Arterial Pressure (MAP)

While maintaining an adequate MAP is essential for organ perfusion, the choice and dose of vasopressors are under increasing scrutiny. There is a growing understanding that systemic blood pressure does not always correlate with micro-mucosal perfusion of the gastric fundus. Current strategies emphasize maintaining an optimal MAP (>65–70 mmHg) while minimizing the alpha-adrenergic burden, as excessive norepinephrine may paradoxically reduce the capillary flow in the most distal (and vulnerable) part of the conduit [20,21,22].

### 3.3. Evolution of Analgesia: Beyond the Epidural Gold Standard

Thoracic epidural analgesia has long been the “gold standard” within ERAS frameworks due to its superior pain control and reduction in pulmonary complications [23]. However, the high incidence of hypotension (20–76%) remains a significant concern for anastomotic perfusion.

To mitigate this, the last five years have seen an increase in the use of alternative regional techniques, such as paravertebral blocks or erector spinae plane blocks. These techniques offer comparable analgesic efficacy to TEA but with a significantly more stable hemodynamic profile and less need for vasopressor support, potentially offering a safer environment for the maturing anastomosis [24].

### 3.4. Integrated Perfusion Monitoring

The most significant update in this section is the integration of anesthesiological management with intraoperative perfusion assessment. The synergy between the anesthesiologist (optimizing cardiac output and MAP) and the surgeon (evaluating the conduit via Indocyanine Green (ICG) fluorescence) is becoming the new standard. This “multidisciplinary hemodynamics” ensures that the technical success of the anastomosis is supported by a stable and optimized physiological environment [25].

To facilitate clinical implementation, key perioperative hemodynamic and analgesic considerations are summarized in Table 1.

## 4. Surgical-Technical Refinements: From Intuition to Objective Assessment

The technical execution of esophagectomy remains the pivoting point where perioperative optimization meets surgical reality. While the fundamental principles of esophageal reconstruction have been established for decades, the last five years have witnessed a definitive shift: the reliance on a surgeon’s “clinical intuition” is increasingly being augmented by objective, real-time data and proactive technological interventions.

### 4.1. Surgical Approach and the Choice of Anastomotic Site

The evolution from open surgery to Minimally Invasive Esophagectomy (MIE) has reached a state of maturity. Large-scale data, including the TIME trial, have demonstrated that MIE achieves at least comparable AL rates to open surgery while significantly reducing overall pulmonary morbidity [26]. Within this framework, the debate regarding the anatomical location of the anastomosis—cervical versus intrathoracic—persists, yet the “roadmap” has become clearer. Historically, cervical anastomoses were favored for their safer management of leaks; however, they remain burdened by higher leak rates (often exceeding 25%) due to increased longitudinal tension and the inherent vulnerability of the poorly perfused gastric fundus [26,27]. Conversely, the intrathoracic approach (Ivor Lewis) allows for a reconstruction under less tension in a better-perfused segment of the conduit. Driven by improved interventional management of intrathoracic complications, there is a clear international trend toward intrathoracic reconstructions as the preferred standard [28].

### 4.2. Conduit Construction: Balancing Geometry and Perfusion

The creation of the gastric conduit is a delicate balance between gaining sufficient length and preserving vascular integrity. The historical debate between utilizing the “whole stomach”—as advocated by Collard [29] and based on the intramural vascular networks described by Levasseur and Couinaud [30,31]—and a narrow gastric tube has largely settled on a “middle ground.” Most contemporary experts utilize a conduit width of approximately 4–5 cm. While a narrow tube may reduce tension, a “narrow but not too narrow” approach preserves the essential vascular arcades along the greater curvature. Technical refinements now focus on a modified incision of the lesser curvature to maintain maximum intramural perfusion while achieving the necessary length. In cases of insufficient mobility, classic maneuvers such as duodenal mobilization (Kocher’s maneuver) remain indispensable tools to ensure a tension-free anastomosis, which is a prerequisite for uneventful healing.

### 4.3. The Technical Execution and Reinforcement of the Anastomosis

Regarding the specific anastomotic design, evidence continues to suggest that the reproducibility of the chosen technique and the surgeon’s specific experience exert a far greater influence on clinical outcomes than the choice of the device or the suturing method itself [28]. While mechanical approaches—particularly circular stapling for intrathoracic and side-to-side linear stapling for cervical reconstructions—have long been the standard due to their technical consistency, the widespread adoption of Robot-Assisted Minimally Invasive Esophagectomy (RAMIE) has introduced a significant shift. The enhanced dexterity and 3D visualization provided by robotic systems have led to a resurgence of hand-sewn intrathoracic anastomoses. What was once a technically demanding maneuver in conventional thoracoscopy has become highly reproducible with robotic assistance [32].

However, despite this “technical renaissance” of the hand-sewn suture, high-level evidence and large-scale registries indicate that this has not yet translated into a superior reduction in AL rates compared to stapled techniques. The “general wisdom” thus remains unchanged: no single technique is demonstrably superior, and the choice of anastomosis should be dictated by the surgeon’s institutional volume and individual proficiency.

To further safeguard the anastomosis, additional reinforcement measures remain a widely endorsed adjunct. The use of a pedicled omental flap is a cornerstone in many high-volume centers; by providing a biological seal and promoting angiogenesis via the secretion of vascular endothelial growth factor (VEGF), it can contain minor defects and prevent the progression to fulminant mediastinitis [33].

As an alternative or complementary maneuver, ‘pleural tenting’—the suturing of a mobilized pleura flap over the completed anastomosis—is frequently employed, particularly in intrathoracic reconstructions. While comparative studies specifically evaluating pleural reinforcement are less abundant than those for omental wrapping, both techniques share the same surgical rationale: creating a well-vascularized barrier to mitigate the septic sequelae of potential leaks. Despite the lack of definitive randomized evidence for a universal reduction in primary leak rates, these flaps are particularly recommended in high-risk scenarios, such as marginal conduit perfusion or following neoadjuvant radiochemotherapy [34]. In addition to mechanical reinforcement techniques, the intraoperative use of surgical sealants and biological glues has been explored as an adjunct to support anastomotic integrity. While these materials may provide a local sealing effect and potentially reduce microleakage, the available evidence remains limited and heterogeneous. Although recent randomized data, such as the PLACE030 trial investigating fibrin sealant in cervical esophagogastric anastomoses, suggest a potential benefit, the findings are not yet sufficient to support routine use across all settings [35]. Therefore, their role should currently be considered selective, particularly in high-risk situations.

### 4.4. Objective Intraoperative Assessment: Validating Perfusion and Mechanical Integrity

**Figure 1:**
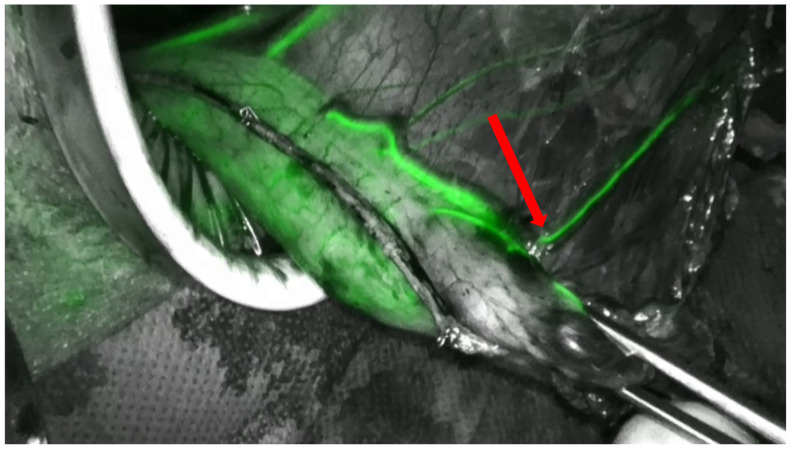
ICG angiogram of the tubulized gastric conduit. The red arrow marks the fluorescing vascular arcade at the greater curvature.

Perhaps the most significant update in the surgical roadmap is the transition from subjective to objective perfusion assessment. Relying on “rosy color” or active bleeding from the staple line has been shown to be notoriously unreliable. Indocyanine Green (ICG) fluorescence imaging has emerged as the new gold standard for real-time visualization of conduit vascularity. By identifying the “watershed area” of reduced perfusion, ICG allows for a precise, data-driven selection of the anastomotic site (Figure 1). Recent meta-analyses confirm that ICG-guided surgery significantly reduces AL rates, and the field is now moving toward quantitative fluorescence analysis and machine learning algorithms to eliminate inter-observer variability and standardize the interpretation of tissue viability. However, vascular viability is only one side of the coin; mechanical integrity represents the other. Complementing perfusion assessment, intraoperative leak testing has gained renewed importance. High-level evidence from a recent umbrella review supports the routine use of integrity tests such as flexible endoscopy, methylene blue injection, or emerging techniques such as intraluminal indocyanine green (ICG) application, which may allow real-time detection of anastomotic defects immediately [36]. These maneuvers (supported by Class II evidence) allow for the preemptive intraoperative repair of minor leaks, thereby significantly reducing the incidence of postoperative AL.

### 4.5. Ischemic Conditioning: Priming the Conduit for Success

The concept of ischemic conditioning—the intentional, partial devascularization of the stomach prior to esophagectomy—remains one of the most debated biological strategies in esophageal surgery. The physiological rationale is based on the “delay phenomenon”: by ligating the left gastric and/or short gastric arteries (either surgically or via interventional embolization) approximately two to three weeks before the definitive resection, the stomach is forced to adapt to a state of relative ischemia. This trigger is thought to stimulate the redistribution of blood flow through the remaining right gastric and gastroepiploic arcades and potentially enhance submucosal microvascular density. Despite its intuitive biological appeal, the clinical translation of ischemic conditioning into a universal reduction in AL rates remains inconsistent. Several randomized controlled trials and subsequent meta-analyses have struggled to demonstrate a significant, broad-based benefit. While some studies suggest a reduction in the severity of leaks and a demarcation of ischemic zones (allowing for a more precise selection of the anastomotic site), others have shown no difference in primary AL rates [37]. Consequently, many high-volume centers have moved away from its routine application. The lack of widespread adoption is also due to practical and technical concerns. The “two-stage” approach required for ischemic conditioning adds cost, requires additional resources (interventional radiology or a prior laparoscopic procedure), and—perhaps most critically—induces peritoneal adhesions. These adhesions can significantly complicate the subsequent gastric mobilization and oncological lymphadenectomy during the main procedure.

Nevertheless, ischemic conditioning maintains a specialized role in the modern “roadmap.” It is increasingly viewed as a rescue or “priming” strategy for high-risk patients—such as those with significant peripheral vascular disease, prior gastric surgery, or individuals undergoing a complex “two-stage” esophagectomy where the conduit’s microcirculation is deemed particularly vulnerable. In these selected cohorts, the biological pre-conditioning of the gastric fundus may provide the critical margin of safety needed to prevent conduit necrosis [38].

### 4.6. Mechanical and Biological Protection: From Decompression to pEVT

Beyond the technical construction of the anastomosis, the immediate postoperative phase requires a strategy to mitigate physical forces that may jeopardize tissue healing. This involves a dual approach: minimizing endoluminal pressure and proactively supporting the healing zone. The physiological integrity of the gastric conduit is highly sensitive to endoluminal pressure. According to Laplace’s law, wall tension in a cylindrical structure increases proportionally with its radius and internal pressure. In the context of esophagectomy, an undrained and dilated gastric conduit results in increased intramural strain, which may compromise microvascular perfusion and oxygen delivery to the vulnerable anastomotic site. To counter this, routine postoperative gastric decompression remains a cornerstone of care. While traditional ERAS protocols often advocate early removal of nasogastric tubes to facilitate recovery and reduce patient discomfort, many specialized centers favor a more nuanced approach. However, recent high-level evidence has challenged the safety of omitting nasogastric decompression. In a large multicenter randomized controlled trial, omission of postoperative nasogastric tube use failed to demonstrate non-inferiority and was associated with a higher rate of anastomotic leakage compared to routine decompression (22.1% vs. 15.2%) [39]. These findings suggest that routine nasogastric decompression may play a protective role and that complete omission should be approached with caution in esophagectomy patients. In this context, some centers combine continuous decompression with early oral intake, allowing patients to drink immediately after surgery while maintaining active suction via a nasogastric tube. Beyond patient comfort, this strategy may confer additional physiological benefits: the continuous “flushing effect” associated with early oral intake may dilute bacterial colonization at the anastomotic site and thereby potentially support anastomotic healing. Although this concept remains insufficiently investigated, it represents a plausible adjunct mechanism within a multimodal prevention strategy.

**Figure 2:**
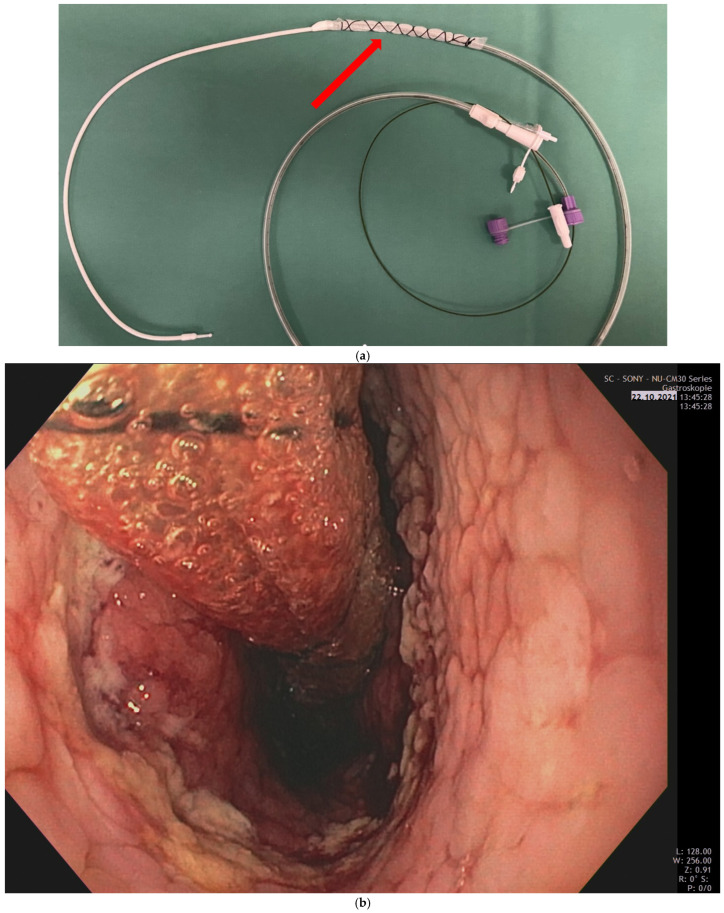
(**a**): Custom-built sump drainage using a Freka Trelumina^®^ (Fresenius Kabi AG, Kriens, Switzerland) wrapped with Suprasorb^®^ CNP Drainage Film (Lohmann & Rauscher GmbH & Co. KG, Neuwied, Germany). (**b**): Endoscopic presentation of the custom-built sump drain placed in the conduit lumen after completion of the esophago-gastric anastomosis.

The use of a double-lumen sump drainage system (e.g., a thin foil probe with continuous suction) allows for reliable decompression without necessitating prolonged fasting (Figure 2a,b). This “suction-safe” environment enables early oral intake of clear liquids while effectively preventing conduit distension and reducing the risk of aspiration, thereby balancing the goals of early mobilization with the requirement for low anastomotic wall tension.

The most disruptive innovation in recent years is the transition of Endoluminal Vacuum Therapy (EVT) from a reactive treatment to a preemptive strategy (pEVT). While EVT is the established gold standard for managing manifest leaks with success rates of 80–90%, pEVT aims to prevent the leak from ever becoming clinically significant. By placing a polyurethane sponge at the anastomotic site intraoperatively and applying continuous negative pressure for the first 3 to 5 postoperative days, pEVT serves several functions: it provides continuous drainage of the anastomotic zone, promotes local granulation, and may improve regional microperfusion. Although results from randomized controlled trials are still awaited to provide Level I evidence, current high-volume center data and propensity-matched analyses are highly promising [40,41]. These studies suggest that pEVT not only reduces the primary incidence of AL in high-risk patients but, perhaps more importantly, significantly attenuates the septic sequelae of minor defects, essentially “downgrading” potential Type II or III leaks to manageable Type I events [42].

## 5. Postoperative Management: Proactive Surveillance and Secondary Prevention

The transition from the operating theater to the surgical ward marks a critical phase where the focus shifts from prevention to vigilant, multi-layered surveillance. In modern esophageal surgery, the postoperative “roadmap” has evolved into a proactive strategy designed to detect subclinical deviations and initiate “failure to rescue” protocols before a minor leak escalates into a catastrophic septic event.

The foundation of contemporary management is the standardized classification provided by the Esophagectomy Complications Consensus Group (ECCG) [1]. By stratifying ALs into three distinct types—ranging from Type I (conservative management) to Type III (surgical reintervention)—the ECCG framework has enabled a more nuanced and reproducible therapeutic approach.

Early detection remains the cornerstone of success, beginning with the systematic monitoring of inflammatory biomarkers. While white blood cell counts and procalcitonin (PCT) provide additional data, C-reactive protein (CRP) has established itself as a vital surrogate for anastomotic integrity. A steady decline in CRP levels during the first postoperative week serves as a reliable indicator of an uneventful course; conversely, a failure to “clear” inflammatory markers should trigger an immediate escalation of the diagnostic cascade, even in the absence of overt clinical symptoms like fever or tachycardia.

The definitive diagnosis then relies on a combination of cross-sectional imaging and direct visualization. Computed Tomography (CT) with intravenous and oral contrast is the primary workhorse, capable of identifying extraluminal air, mediastinal collections, and the “biological health” of the gastric conduit. However, because early CT (POD 1–3) can be difficult to interpret due to post-surgical changes, endoscopy has emerged as the definitive gold standard [43]. The historical fear that early endoscopy might mechanically jeopardize a fresh anastomosis has been refuted; today, it is considered a safe and essential tool for the direct assessment of mucosal integrity and the precise grading of defects. While the contrast swallow study (fluoroscopy) remains a traditional fixture, its role has become more specialized, often reserved for stable patients to confirm functional transit rather than as a primary screening tool for occult leaks.

### ERAS and the Biology of Healing

ERAS pathways support the biological environment of the anastomosis through early functional optimization. Central to this is the paradigm of early enteral nutrition, typically initiated via a jejunostomy within 24 h of surgery [23]. This maintains gut barrier function and provides the metabolic substrates necessary for tissue repair without increasing the risk of leakage. A critical, often overlooked component of the postoperative phase is pharmacological management. While multimodal analgesia is a cornerstone of ERAS, the choice of agents requires caution. Current evidence identifies the use of non-steroidal anti-inflammatory drugs (NSAIDs) as a significant modifiable risk factor [37]. Non-selective NSAIDs, such as diclofenac, have been associated with a more than threefold increase in AL risk (OR 3.02), likely due to their detrimental effects on microvascular blood flow and collagen synthesis. Consequently, postoperative pain management should prioritize selective COX-2 inhibitors or opioid-sparing concepts that avoid non-selective NSAIDs to safeguard the maturing anastomosis. Furthermore, maintaining the “hemodynamic tightrope” established intraoperatively remains crucial; avoiding interstitial edema through judicious fluid management is as vital to anastomotic maturation as ensuring adequate microvascular perfusion.

## 6. Discussion and Future Perspectives

Five years after our initial review of this landscape, AL remains the defining “Achilles’ heel” of esophageal surgery. However, the paradigm has shifted significantly. While the 2021 perspective was characterized by a certain empiric pragmatism and a lack of standardized evidence, we have now entered an era of data-driven precision. The transition from purely technical modifications to a comprehensive, multidisciplinary “bundle” approach represents the most substantial evolution in recent years.

The importance of AL prevention extends far beyond the immediate postoperative recovery. Emerging evidence now recognizes AL as a significant negative predictor of long-term oncological outcomes [4,44]. The profound systemic inflammatory response triggered by a leak—potentially coupled with a delay in adjuvant therapy—appears to create a biological environment conducive to tumor cell proliferation. Patients suffering from a leak face significantly worse overall and disease-free survival rates compared to those with an uncomplicated course. Furthermore, the functional “cost” is substantial, encompassing persistent dysphagia, chronic stricture formation, and significant psychological distress. These findings reinforce the central thesis of this update: effective management of the “Achilles’ heel” must be a robust, multimodal prevention and early detection strategy.

The contemporary roadmap for AL prevention is built on three pillars (Table 2). Preoperatively, we have moved beyond chronological age to assess physiological resilience through objective markers like CT-based sarcopenia assessment and targeted prehabilitation. Intraoperatively, the subjective “clinical eye” is increasingly augmented by real-time perfusion imaging via ICG and technical refinements that balance conduit geometry with microvascular integrity. Postoperatively, the proactive use of pEVT and standardized biomarker surveillance (e.g., CRP kinetics) has transformed our ability to “rescue” patients before a minor leak escalates into a catastrophic event.

Looking ahead, the roadmap points toward a personalized, data-driven era. Machine learning algorithms that integrate preoperative sarcopenia scores, intraoperative ICG perfusion kinetics, and postoperative biomarker trends are being developed to provide individual risk assessments in real-time. Such tools, combined with emerging technologies like hyperspectral imaging [20] and novel bioengineered tissue sealants [45], promise to further standardize care and minimize the impact of this major surgical challenge.

Yet, technological innovation alone is not a panacea. The “human factor” remains paramount. The long learning curve of minimally invasive esophagectomy and the clear association between institutional volume and outcomes underscore the necessity of surgical centralization, specialized training curricula, and commitment to standardized reporting. By combining surgical expertise with evidence-based technological adjuncts across all perioperative phases, we can continue to refine our roadmap and further improve outcomes for patients undergoing esophagectomy for cancer. Despite their promise, the implementation of emerging technologies such as machine learning models, quantitative perfusion analysis, and hyperspectral imaging faces several challenges. These include the need for standardized data acquisition, external validation across centers, limited generalizability, and issues related to interpretability and integration into clinical workflows. Addressing these barriers will be essential before widespread clinical adoption can be achieved.

## 7. Conclusions

Prevention of anastomotic leakage in esophageal surgery has evolved from technique-focused approaches toward a comprehensive, multimodal strategy spanning the entire perioperative pathway. This contemporary roadmap integrates preoperative patient optimization, objective intraoperative perfusion assessment, and proactive postoperative management, with the aim of improving both short-term recovery and long-term oncological outcomes.

Looking ahead, advances in imaging technologies and machine learning may further support individualized risk assessment and more precise surgical decision-making. However, technological innovation cannot replace surgical expertise; the “human factor” remains essential, underscoring the need for centralization and specialized training. By combining technical skill with structured, evidence-based care, further improvements in patient safety, survival, and quality of life can be achieved.

## Figures and Tables

**Table 1:** Practical perioperative hemodynamic and analgesic considerations in esophagectomy.

Domain	Recommendation	Rationale
Mean Arterial Pressure (MAP)	Maintain MAP ≥ 65–70 mmHg	Ensures adequate organ and conduit perfusion while avoiding hypoperfusion
Fluid Management	Use goal-directed fluid therapy (GDT) guided by dynamic parameters (e.g., stroke volume variation)	Avoids both hypovolemia-induced ischemia and fluid overload-related edema
Vasopressor Use	Minimize high-dose vasopressors; use lowest effective dose	Excessive alpha-adrenergic stimulation may impair microvascular perfusion of the gastric conduit
Perfusion Monitoring	Integrate systemic hemodynamics with intraoperative ICG assessment	Aligns macro-hemodynamic stability with real-time evaluation of conduit perfusion
Analgesia Strategy	Consider alternatives to thoracic epidural (e.g., paravertebral or erector spinae plane blocks) in selected patients	Reduces risk of hypotension while maintaining effective pain control
Multidisciplinary Coordination	Close collaboration between surgical and anesthesiology teams	Optimizes intraoperative decision-making and postoperative outcomes

**Table 2:** Key Take-Home Messages for AL Prevention.

Phase	Strategic Domain	Key Recommendations & Findings
**Preoperative**	Risk Stratification	Move from chronological age to physiological resilience using CT-based sarcopenia assessment.
	Prehabilitation	Implement 4–6 week multimodal programs (exercise, nutrition, psychological support) to optimize physiological reserve.
	Metabolic Control	Target HbA1c < 7–8% and provide 7–14 days of nutritional support for malnourished patients.
**Intraoperative**	Perfusion Monitoring	Use Indocyanine Green (ICG) fluorescence angiography as the gold standard for objective anastomotic site selection.
	Conduit Design	Maintain a “middle-ground” conduit width of 4–5 cm to balance microvascular integrity and tension.
	Integrity Testing	Routinely use intraoperative leak tests (endoscopy or methylene blue) to identify and repair technical defects.
	Fluid Management	Prioritize Goal-Directed Fluid Therapy (GDT) and avoid excessive vasopressors to maintain conduit microcirculation.
**Postoperative**	Surveillance	Monitor CRP kinetics; failure to clear inflammatory markers by the first week requires early CT or endoscopy.
	Prevention Tools	Use preemptive endoluminal vacuum therapy (pEVT) in high-risk cases to prevent leaks and mitigate sepsis.
	Pharmacology	Avoid non-selective NSAIDs (e.g., diclofenac), which are associated with a threefold increase in AL risk.
	ERAS	Standardize early enteral nutrition (within 24 h) to maintain gut barrier function.

## Data Availability

No new data were created or analyzed in this study.
